# Urgent and emergency tracheostomy in head and neck cancer: practical guide from the Brazilian Society of Surgical Oncology (SBCO)^☆^

**DOI:** 10.1016/j.bjorl.2026.101865

**Published:** 2026-07-27

**Authors:** Carlos Eduardo Santa Ritta Barreira, Terence Pires de Farias, Fernando Luiz Dias, Roberto Rego Monteiro de Araujo Lima, Izabella Costa Santos, Stenio Roberto de Castro Lima Santos, Heládio Feitosa, Rodrigo Nascimento Pinheiro, Ranna Gabriela Sala, Roberta Oliveira de Almeida, Viviane Rezende de Oliveira, Alexandre Ferreira Oliveira, Paulo Henrique de Sousa Fernandes, Luiz Paulo Kowalski

**Affiliations:** a Hospital DF STAR ‒ Cirurgia de Cabeça e Pescoço, Brasília, DF, Brazil; b Instituto Nacional de Câncer, Seção de Cirurgia de Cabeça e Pescoço (INCA/RJ), Rio de Janeiro, RJ, Brazil; c Universidade Federal do Maranhão (UFMA), Departamento de Medicina I, São Luiz, MA, Brazil; d Instituto do Câncer do Ceará (ICC), Departamento de Cirurgia Oncológica, Fortaleza, CE, Brazil; e Hospital de Base do Distrito Federal, Brasília, DF, Brazil; f Hospital DF STAR, Brasília, DF, Brazil; g Hospital das Forças Armadas, Ministério da Defesa do Brasil, Brasília, DF, Brazil; h Universidade Federal de Juiz de Fora, Departamento de Cirurgia, Juiz de Fora, MG, Brazil; i Faculdade de Medicina da Universidade Federal de Uberlândia, Departamento de Cirurgia Oncológica, Uberlândia, MG, Brazil; j Faculdade de Medicina da Universidade de São Paulo, Departamento de Cirurgia de Cabeça e Pescoço, São Paulo, SP, Brazil; k Universidade de Brasília, DF, Brazil

**Keywords:** Tracheostomy, Head and neck neoplasms, Airway obstruction, Oncologic emergency, Airway management

## Abstract

**Objective:**

Propose a structured decision-making model for the indication of urgent and emergency tracheostomy in patients with head and neck tumors, integrating clinical warning signs, functional airway assessment, and contextual factors, thereby promoting timely indication of the procedure and reducing the morbidity and mortality associated with delayed emergency interventions.

**Methods:**

This guideline was developed through consensus by the Head and Neck Neoplasms Commission of the Brazilian Society of Surgical Oncology (SBCO). A systematic critical review of the literature was conducted using the MEDLINE and SciELO databases, prioritizing studies with high or moderate levels of evidence. Recommendations were formulated and classified according to the GRADE system, reflecting both the strength of recommendation and the quality of available evidence, with adaptation to the Brazilian healthcare context.

**Results:**

Recommendations are presented based on an integrated assessment of clinical, endoscopic, and contextual criteria for the indication of urgent and emergency tracheostomy. Particular emphasis is given to the concept of a “window of opportunity” before airway collapse, the preferred use of awake tracheostomy under local anesthesia in patients at substantial risk of acute airway obstruction, prioritizing awake endoscopic intubation in anaplastic thyroid carcinoma, and the careful strategic selection of the tracheostomy level in laryngeal neoplasms, considering subsequent oncologic surgical treatment.

**Conclusion:**

An anticipatory, integrated, and strategically planned approach to tracheostomy indication in patients with head and neck tumors may reduce catastrophic respiratory events, preserve the oncologic treatment pathway, and improve patient safety, despite the limitations inherent to the currently available evidence.

## Highlights

• Consensus-based guidance for urgent/emergency tracheostomy in head and neck cancer.
• Clinical, endoscopic, and contextual criteria guide airway decision-making.
• Early airway “window of opportunity” recognition prevents catastrophic obstruction.
• Awake tracheostomy under local anesthesia is preferred in high-risk patients.
• Tracheostomy planning should preserve the subsequent oncologic treatment strategy.

## Introduction

Tracheostomy constitutes an essential procedure for definitive airway management in patients with head and neck neoplasms at imminent risk of airway obstruction. These situations are potentially fatal and demand rapid and precise clinical decision-making, with direct implications for both quality of life and the prognosis of the underlying disease.

Classical guidelines for tracheostomy indication focus primarily on non-oncological settings, such as prolonged mechanical ventilation and respiratory failure in intensive care units. In specialized oncology centers, however, tracheostomy is frequently indicated to manage respiratory complications resulting from tumors of the oral cavity, oropharynx, hypopharynx, larynx, anaplastic thyroid carcinoma, and other large cervical masses. Such tumors may cause progressive or sudden narrowing of the upper airway, along with relevant functional impariments such as dysphagia, sialorrhea, recurrent aspiration, reduced oral opening, and impairment of laryngeal protection.

Such scenarios are marked by high clinical stress, physiological instability, and an increased risk of severe complications, including hypoxia and cardiorespiratory arrest. Despite this, a notable scarcity of specific practical guidelines addressing airway management in oncologic urgent and emergency scenarios persists. In Brazil, approximately 80% of head and neck tumors present at or enter treatment in advanced stages, according to data from the National Cancer Institute (INCA).^1^ Consequently, emergency tracheostomy is indicated more frequently. Data from Hospital de Câncer I of INCA (Rio de Janeiro) for 2025 document 120 tracheostomies preformed, of which 66 were elective as part of oncologic treatment, 24 were related to prolonged mechanical ventilation in the ICU, and 30 were performed on an urgent basis. These descriptive, non-comparative data serve to illustrate the clinical relevance of the topic.

Reported complication rates associated with tracheostomy vary widely, potentially exceeding 40%–50% when minor and major events are combined, especially in emergency scenarios and in the absence of experienced teams.^2^^,^^3^ This risk is further compounded when the procedure is performed under adverse technical conditions, without adequate planning, or in an uncontrolled environment.

Although cricothyroidotomy is traditionally indicated as the primary surgical airway access procedure in situations of extreme emergency, its application in this patient population presents significant limitations. Tumors of the pharynx and larynx may involve the cricothyroid region, rendering high airway access susceptible to tumor transgression and neoplastic implantation along the surgical tract, with a potential negative impact on local disease control and subsequent treatment. Furthermore, previous radiotherapy frequently alters tissue integrity, increasing the risk of technical failure and perioperative complications. Given that airway obstruction in these patients is generally progressive and sustained, cricothyroidotomy does not adequately provide definitive airway control and should be reserved for life-threatening emergencies requiring immediate intervention.

Several authors have proposed scoring systems to assist in the indication of tracheostomy in head and neck oncologic surgery, incorporating clinical, tumor-related, and surgical variables.^4^^,^^5^ However, none of these scores was developed or validated for urgent oncologic scenarios and none has demonstrated the capacity to replace individualized clinical judgment. In practice, airway management in these situations relies on the integration of dynamic clinical assessment, endoscopic findings, tumor characteristics, and the experience of the treating team.

In view of these gaps, this practical guide was developed to support decision-making regarding the indication and performance of urgent and emergency tracheostomy in patients with head and neck tumors, in a clear and clinically applicable manner.

## Methods

This guideline was developed by the Head and Neck Neoplasms Commission of the Brazilian Society of Surgical Oncology (SBCO) to propose a consensus-based structured decision-making model for the indication and performance of urgent and emergency tracheostomy in oncologic patients.

Recommendations were formulated using the GRADE system (Grading of Recommendations Assessment, Development and Evaluation), which classifies guidance according to the strength of the recommendation (strong or weak) and the quality of the evidence (high, moderate, low, or very low). Strong recommendations are expressed as “recommendation,” whereas weak recommendations are expressed as “suggestion”.^6^

A critical analysis of the available literature was performed using the MEDLINE and SciELO databases, prioritizing studies with high or moderate levels of evidence, in addition to recognized international consensus statements and guidelines, adapted to the conditions of the Brazilian healthcare context.

## Recommendations for urgent and emergency tracheostomy in head and neck tumors

The indication for tracheostomy in patients with head and neck tumors should be based on an integrated assessment of clinical, endoscopic, and contextual criteria, aiming to prevent potentially fatal acute respiratory events and preserve the oncologic treatment pathway.

The distinction between urgent and emergency tracheostomy is fundamental. Emergency tracheostomy is performed in the setting of imminent or established airway failure, often under adverse technical conditions and associated with higher morbidity. In contrast, urgent tracheostomy is performed in the context of progressive airway compromise, still within a window of opportunity that allows appropriate planning, a controlled environment, and lower technical risk.

## Clinical criteria

Recommendation 1 ‒ Immediate tracheostomy is strongly recommended in the presence of any major clinical sign of threatened airway, including: inspiratory stridor at rest; progressive dyspnea with use of accessory muscles; orthopnea or inability to tolerate the supine position; desaturation or signs of hypoventilation; altered level of consciousness attributable to hypoxia; and tumor bleeding with risk of aspiration or obstruction.

Justification: These findings indicate imminent risk of acute respiratory failure and are associated with higher rates of failure of conventional airway management, making tracheostomy the safest strategy for definitive airway maintenance.^2^^,^^7^, ^8^, ^9^ (GRADE: Strong Recommendation, moderate quality of evidence)

Suggestion 1 ‒ Special attention is suggested for patients with minor but progressive or combined clinical features, such as progressive dysphonia, dysphagia with sialorrhea, recurrent choking episodes carrying risk of bronchoaspiration, reduced oral opening, and exertional dyspnea, supporting anticipatory tracheostomy indication.

Justification: Progression of these signs increases the risk of sudden airway deterioration; elective intervention may thus be protective against higher-risk emergency scenarios.^8^^,^^10^ (GRADE: Suggestion, low to moderate quality of evidence)

## Endoscopic criteria

While no studies support tracheostomy indication based solely on isolated endoscopic criteria, serial endoscopic findings, serial endoscopic evaluation of the upper airway is an essential component in assessing airway management safety. Findings such as significant luminal narrowing, structural instability, post-radiotherapy edema, vocal fold fixation, mobile masses prone to inspiratory collapse, or friable tumors with recurrent bleeding all represent indicators of increased risk.

Suggestion 2 ‒ Tracheostomy should be considered in the presence of endoscopic findings indicating significant structural compromise of the airway, especially when combined with clinical or contextual risk factors.

Justification: Endoscopic findings considered to carry higher risk include tumor progression with reduction of functional airway space; significant edema during or after radiotherapy; unilateral or bilateral vocal fold fixation associated with tumor volume; presence of a mobile tumor mass with potential for inspiratory collapse; and friable tumors with recurrent bleeding and risk of bronchoaspiration.^8^^,^^9^^,^^11^ (GRADE: Suggestion, low to moderate quality of evidence)

## Contextual criteria

Recommendation 2 ‒ Tracheostomy is strongly recommended as an airway protection measure when contextual factors increase the risk of sudden airway failure, including rapid symptom progression, anticipated failure of orotracheal intubation, imminent need for extensive surgery, prior radiotherapy with persistent edema, or unavailability of an experienced team or advanced airway resources.

Justification: In these scenarios, tracheostomy reduces dependence on unplanned emergency interventions and mitigates the risk of hypoxia, aspiration, and cardiorespiratory arrest.^8^^,^^9^^,^^12^ (GRADE: Strong Recommendation, moderate quality of evidence)

Recommendation 3 ‒ The indication for urgent or emergency tracheostomy should be based on the combined assessment of clinical, endoscopic, and contextual criteria, rather than on isolated application of existing scoring systems.

Justification: Progressive respiratory deterioration, associated with clinical signs, dynamic endoscopic findings, and adverse contextual factors, represents a period in which the airway can still be controlled in a planned manner, within an appropriate environment and with lower technical risk. Delaying the decision until the onset of acute respiratory failure shifts the intervention to an emergency scenario, often characterized by unfavorable conditions, higher complication rates, and increased morbidity and mortality. An integrated approach therefore enables more precise decision-making, promotes timely procedure indication, and reduces morbidity and mortality associated with tracheostomies performed under conditions of imminent airway collapse.^8^^,^^9^^,^^13^^,^^14^ (GRADE: Strong Recommendation, moderate quality of evidence)

Recommendation 4 ‒ Awake tracheostomy under local anesthesia is strongly recommended in patients at high risk of acute airway obstruction, characterized by progressive anatomical obstruction, tumor bleeding, significant cervical distortion, or low predictability of safe orotracheal intubation.

Justification: Patients with head and neck tumors frequently exhibit tumor progression, inflammatory edema, local bleeding, and functional alterations of the larynx and swallowing, which may lead to rapid and unpredictable respiratory deterioration. Classical difficult airway guidelines, although not specific to oncology, recognize that progressive anatomical obstruction and cervical distortion make repeated intubation attempts particularly dangerous. Repeated intubation attempts, even with the aid of advanced techniques such as endoscopic intubation or nasofibrolaryngoscopy, should be avoided when the risk of failure is high, as they substantially increase the probability of hypoxia, ventilatory failure, and cardiorespiratory arrest. In selected scenarios, awake endoscopic intubation may be considered, provided that it is performed by an experienced team, in a controlled environment, and without delaying definitive surgical airway access.^8^, ^9^, ^10^ (GRADE: Strong Recommendation, moderate quality of evidence)

Recommendation 5 ‒ In patients with anaplastic thyroid carcinoma and large cervical masses causing significant tracheal displacement, awake endoscopic intubation is strongly recommended as the initial strategy whenever feasible and when performed by an experienced team, while routine prophylactic tracheostomy is not recommended.

Justification: In these scenarios, anatomical identification of the airway by isolated clinical methods becomes imprecise, and endoscopic intubation provides greater visual control and predictability of success. Attempts at surgical airway access without adequate anatomical reference should be avoided, as tracheal displacement, tumor infiltration, and distortion of cervical planes significantly increase the risk of false passage, bleeding, and ventilatory failure. The prior use of imaging studies contributes to spatial orientation of the trachea and reduction of adverse events, aligning with safety principles in the management of complex oncologic airways. (GRADE: Strong Recommendation, moderate quality of evidence)

Recommendation 6 ‒ In laryngeal neoplasms, the level of tracheostomy should be strategically defined according to the subsequent oncologic surgical planning, with clear distinction between temporary and definitive tracheostomy.

Justification: In laryngeal neoplasms, tracheostomy should not be regarded solely as a rescue measure for airway management, but rather as an integral part of the overall oncologic strategy. Inappropriate selection of the stoma level may compromise future options for laryngeal preservation or hinder the execution of radical surgery. From the perspective of conservative laryngeal surgery, when tracheostomy is temporary, a low tracheostomy should preferably be performed, positioned between the lower rings of the cervical trachea, in order to avoid interference with laryngeal preservation procedures such as cricohyoidopexy.^15^, ^16^, ^17^, ^18^ (GRADE: Strong Recommendation, moderate quality of evidence)

## Conclusion

Tracheostomy in patients with head and neck neoplasms should be regarded not only as an immediate airway rescue intervention but also as a strategic decision with direct implications for quality of life, continuity of oncologic treatment, and overall patient prognosis.

An anticipatory approach, grounded in the integrated evaluation of clinical, endoscopic, and contextual criteria, enables reduction of catastrophic respiratory events, minimization of emergency interventions under unfavorable technical conditions, and preservation of the oncologic therapeutic trajectory.

Timely recognition of the window of opportunity for tracheostomy indication represents a key element of patient safety and good clinical practice in head and neck oncology.

Despite the inherent ethical and logistical constraints of urgent and emergency clinical scenarios, which preclude the availability of high-quality randomized evidence, the proposed model represents a practical, applicable, and clinically relevant framework amenable to further validation through prospective studies and implementation across diverse healthcare settings.

## ORCID ID

Carlos Eduardo Santa Ritta Barreira: 0000-0002-2126-1811

Terence Pires de Farias: 0000-0001-7287-5736

Fernando Luiz Dias: 0000-0003-1000-7436

Roberto Rego Monteiro de Araujo Lima: 0000-0001-5730-0707

Izabella Costa Santos: 0000-0002-6426-2419

Stenio Roberto de Castro Lima Santos: 0009-0007-9910-916X

Heládio Feitosa: 0000-0001-6455-916X

Rodrigo Nascimento Pinheiro: 0000-0002-2715-7628

Ranna Gabriela Sala: 0009-0008-2509-9883

Ro berta Oliveira de Almeida: 0009-0007-3187-3989

Viviane Rezende de Oliveira: 0000-0001-8041-3689

Alexandre Ferreira Oliveira: 0000-0002-7500-6752

Paulo Henrique de Sousa Fernandes: 0000-0001-5476-2017

Luiz Paulo Kowalski: 0000-0002-0481-156X

## Synopsis

This consensus guideline from the Brazilian Society of Surgical Oncology provides evidence-based recommendations for the indication and planning of urgent and emergency tracheostomy in patients with head and neck neoplasms. It emphasizes anticipatory assessment, timely intervention before airway collapse, and technical decision-making aligned with both immediate airway safety and subsequent oncologic management.

## Authors' contributions

Carlos Eduardo Santa Ritta Barreira: Conceptualization (equal); project administration (equal); writing-original draft (equal); writing-review & editing (equal).

Terence Pires de Farias: Conceptualization (equal); project administration (equal); writing-original draft (equal); writing-review & editing (equal).

Fernando Luiz Dias: Conceptualization (equal); project administration (equal); writing-original draft (equal); writing-review & editing (equal).

Roberto Rego Monteiro de Araujo Lima: Writing-original draft (equal); writing-review & editing (equal).

Izabella Costa Santos Writing-original draft (equal); writing-review & editing (equal).

Stenio Roberto de Castro Lima Santos: Writing-original draft (equal); writing-review & editing (equal).

Heládio Feitosa: Writing-original draft (equal); writing-review & editing (equal).

Rodrigo Nascimento Pinheiro: Writing-original draft (equal); writing-review & editing (equal).

Ranna Gabriela Sala: Writing-original draft (equal); writing-review & editing (equal).

Roberta Oliveira de Almeida: Writing-original draft (equal); writing-review & editing (equal).

Viviane Rezende de Oliveira: Writing-original draft (equal); writing-review & editing (Equal).

Alexandre Ferreira Oliveira: Conceptualization (equal); project administration (equal); writing-original draft (equal); writing-review & editing (equal).

Paulo Henrique de Sousa Fernandes: Project administration (equal); writing-original draft (equal); writing-review & editing (equal).

Luiz Paulo Kowalski: Writing-original draft (equal); writing-review & editing (equal).

## Data availability statement

The authors declare that all data are available in repository.

## Declaration of competing interest

The authors declare that they have no known competing financial interests or personal relationships that could have appeared to influence the work reported in this manuscript.

All authors certify that they have no affiliations with or involvement in any organization or entity with any financial interest (such as honoraria, educational grants, participation in speakers’ bureaus, consultancy, employment, stock ownership, or other equity interest) or non-financial interest (such as personal or professional relationships, academic competition, or intellectual passion) in the subject matter or materials discussed in this manuscript.

Furthermore, no external funding or sponsorship influenced the design, analysis, interpretation, or writing of this work. This manuscript does not include material from other copyright sources, nor does it contain illustrations in which any person could be recognized.
